# SLiMScape: a protein short linear motif analysis plugin for Cytoscape

**DOI:** 10.1186/1471-2105-14-224

**Published:** 2013-07-15

**Authors:** Kevin T O’Brien, Niall J Haslam, Denis C Shields

**Affiliations:** 1 UCD Conway, Institute of Biomolecular and Biomedical Sciences, University College Dublin, Dublin, Ireland; 2 UCD Complex and Adaptive Systems Laboratory, University College Dublin, Dublin, Ireland; 3 School of Medicine and Medical Science, University College Dublin, Dublin, Ireland

## Abstract

**Background:**

Computational protein short linear motif discovery can use protein interaction information to search for motifs among proteins which share a common interactor. Cytoscape provides a visual interface for protein networks but there is no streamlined way to rapidly visualize motifs in a network of proteins, or to integrate computational discovery with such visualizations.

**Results:**

We present SLiMScape, a Cytoscape plugin, which enables both de novo motif discovery and searches for instances of known motifs. Data is presented using Cytoscape’s visualization features thus providing an intuitive interface for interpreting results. The distribution of discovered or user-defined motifs may be selectively displayed and the distribution of protein domains may be viewed simultaneously. To facilitate this SLiMScape automatically retrieves domains for each protein.

**Conclusion:**

SLiMScape provides a platform for performing short linear motif analyses of protein interaction networks by integrating motif discovery and search tools in a network visualization environment. This significantly aids in the discovery of novel short linear motifs and in visualizing the distribution of known motifs.

## Background

High throughput experiments have greatly increased the number of known protein-protein interactions in the human interactome. However, most of these experiments do not indicate the mechanism of interaction. Many of these interactions occur between large globular domains but an estimated 15 - 40% are mediated by functional microdomains [1], of 3 - 10 amino acids in size called Short Linear Motifs (SLiMs). These often occur in intrinsically disordered regions and are involved in a number of functions such as binding, cleavage, subcellular targeting and post translational modifications.

Several predictive tools using many different methods have been developed to identify SLiMs [2-8]. We have developed SLiMScape, a plugin which uses two of these tools to add SLiM discovery and search functionality to Cytoscape [9]. Both novel and known motifs can be detected, providing a powerful tool for exploration and analysis of SLiMs within a protein interaction network. Cytoprophet [10], another Cytoscape plugin, predicts domain and protein interaction networks however SLiMScape is the first to focus on SLiMs.

## Implementation

SLiMScape has been tested on Cytoscape version 2.8.3 and on all major platforms (Linux, Windows and MacOS). It is published under the GNU Lesser General Public License version 3.0. SLiMscape depends on several websites such as the SMART [11] domain database, Uniprot [12] protein database, DBFetch [13] and custom SLimFinder [2] and SLiMSearch [5] web services available at bioware.ucd.ie. For this reason web access is required. It is also possible to run SLiMFinder and SLiMSearch locally, however this requires installation of several other tools. A tutorial is available at bioware.ucd.ie/slimscape.

## Results and discussion

SLiMscape consists of two primary analysis tools. The first, SLiMFinder [2], is capable of finding novel motifs by analyzing a protein interaction network. SLiMFinder has previously been demonstrated as a suitable tool for mining the human interactome for SLiM mediated interactions [14]. The second, SLiMSearch [5] can find new instances of known or potential motifs within the same network. An important feature is the visualization of results within a protein interaction network and in the context of domains in nearby proteins.

### Novel motif discovery

**Figure 1:**
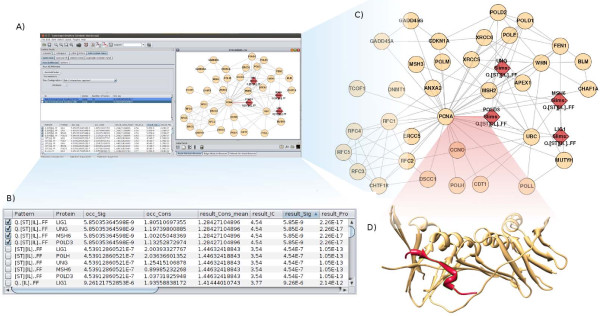
**SLiMScape Interface. A**. The SLiMScape interface displaying SLiMFinder results for the true positive motif LIG_PCNA, a SLiM which binds to Proliferating Cell Nuclear Antigen (PCNA). **B**. The results panel showing several highly significant results from this protein set. **C**. A network view showing SLiMs (red) and other nodes (beige). **D**. A structural view of the SLiM mediated binding between POLD3 (red) and PCNA (beige) via LIG_PCNA. This structural view is provided for example and is not part of the plugin.

SLiMScape uses SLiMFinder to locate statistically over-represented sequences within sets of proteins, defined by selecting subsets of a protein interaction network. A variety of node selection methods are provided to automatically select protein sets and different types of motif can be targeted depending on this selection process. For example, SLiM-mediated interactions can be discovered using the “Batch Interactions” selection method. This will iteratively create protein sets containing each protein (hub) and its interactors (spokes). Figure 1 shows the results of this method on a network consisting of Proliferating Cell Nuclear Antigen (PCNA) and its interactors. Since, in a large network, this can be computationally demanding selection methods include carrying out a single search across a set of selected nodes, specified interactively through Cytoscape’s interface. Another selection method named “Attribute” creates protein sets based on a user-defined attribute. A useful example is subcellular location, which can be used to find subcellular targeting motifs.

The “Batch Domains” selection method creates sets of proteins which contain a particular domain, while “Batch Domains Interactions” creates sets of proteins which interact with a particular domain. This is useful when searching for self interacting proteins and other sequences which bind to a domain. Protein sets can also be created manually, allowing for many custom strategies for finding motifs.

### Known motif search

New instances of a motif can be located using regular expression matching. This is especially useful when trying to find new instances of a SLiM found using SLiMFinder and can also be used to search for a user defined SLiM. Disorder and conservation masking are used to reduce the number of false positives. This functionality is implemented using SLiMSearch.

### Visualization of results

SLiMScape presents an interactive view of results within a protein interaction network. SLiMs are first presented in a table and when activated all instances of that SLiM are highlighted, thus displaying the distribution of the SLiM. Additionally, domain annotations are provided to indicate possible SLiM-domain interactions and aid in the interpretation of results.

## Conclusion

SLiMScape integrates SLiM discovery tools with Cytoscape, a visualization program popular in the field of bioinformatics. This provides a platform for SLiM discovery which allows discovery of novel and known motifs within a protein interaction network. This increases the speed at which SLiM analyses can be performed and aids in the interpretation of results.

## Availability and requirements

• **Project name:** SLiMScape

• **Project home page:**http://sourceforge.net/projects/slimscape/

• **Operating system(s):** Platform independent

• **Programming language:** Java, Python

• **Other requirements:** Cytoscape 2.8.3, Java 1.6 or higher, python 2.7 (optional), Slimsuite (optional), Blast (optional), IUPred (optional), Muscle (optional) and Clustal (optional).

• **License:** GNU LGPLv3

• **Any restrictions to use by non-academics:** see license

## Competing interests

The authors declare that they have no competing interests.

## Authors’ contributions

KOB proposed the idea in collaboration with NH and DS. KOB developed the application. NH and DS provided extensive testing of the application and also provided feedback. DS supervised the project. All authors wrote and approved the final manuscript.
